# Strategies for the Molecular Classification of Medulloblastoma

**DOI:** 10.3390/biomedicines13122845

**Published:** 2025-11-21

**Authors:** Josselen Carina Ramírez-Chiquito, Sergio Juárez-Méndez

**Affiliations:** 1 Experimental Oncology Laboratory, National Institute of Pediatrics, Mexico City 04530, Mexico; 2 Postgraduate in Biological Sciences, Postgraduate Unit, Building D, 1st Floor, Postgraduate Circuit, University City, Coyoacán, Mexico City 04510, Mexico; 3 Molecular Pathology Laboratory, Department of Pathology, National Institute of Pediatrics, Mexico City 04530, Mexico

**Keywords:** cerebellar neoplasms, molecular biology, molecular pathology, molecular diagnostic techniques

## Abstract

Fifteen years ago, an omic study of medulloblastoma revealed the existence of four groups that are biologically and clinically different. Since then, various molecular classification methods have emerged, each with different diagnostic capabilities. Knowledge of these tools is essential for countries with low economic resources to be able to access the technology that is most within their reach since, at present, the precision of molecular classification is crucial for clinical practice, not only because of its relevance in the prognosis of patients but also because of its decisive impact on therapeutic strategies, which are essential for reducing mortality from this disease.

## 1. The Molecular Classification of Medulloblastoma Is the Most Important Prognostic Factor for This Disease

Medulloblastoma (MB) is an embryonic tumor that develops in the posterior cerebellar fossa. It is the second most common type of tumor in children worldwide, with a survival rate ranging from 50% to 80% [1,2,3]. This type of cancer is characterized by high intra- and intertumor heterogeneity, which explains why different tumor entities have been identified over time. Currently, the WHO recognizes four major molecular groups (WNT-activated, SHH-activated: divided on the basis of TP53 mutational status, Group 3 and Group 4) that confer different clinical and prognostic characteristics to patients [4]. More recently, the addition of epigenomic characteristics revealed the existence of 12 subgroups (two for WNT, four for SHH, three for G3 and three for G4) whose prognostic impact is still under study [5,6].

In different populations, the association between the molecular group and the probability of survival has been demonstrated, with WNT medulloblastomas having the best prognosis, followed by SHH and Group 4 (G4) with an intermediate prognosis, and Group 3 (G3) having the worst prognosis [7,8,9,10,11,12,13]. In contrast to the classification at the histological level, only one of the four types (anaplastic large cell medulloblastomas) has been associated with poor patient prognosis [14]. However, this histological type represents only 5–10% of all tumors [8,10,15,16,17].

**Figure 1:**
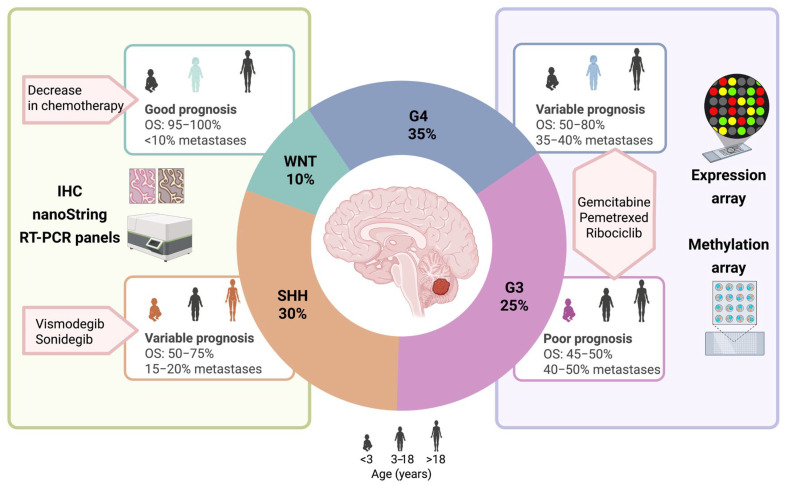
Biological heterogeneity of medulloblastoma and its implications for clinical practice. The diagram represents the clinical diversity of MB molecular groups (frequency, age of presentation, and prognostic characteristics: metastasis and overall survival). It also shows the new therapeutic approaches proposed on the basis of the risk of the molecular group (orange labels). The most accurate classification methods for identifying each group are shown in the green and purple boxes, respectively. OS: 5-year overall survival. IHC: immunohistochemistry.

Currently, the classification of medulloblastoma is carried out in an integral way and includes the clinical, histological and molecular characteristics of each patient. This is the best risk stratification tool and, consequently, the best method for the selection of the ideal treatment for each patient. Therefore, multiple clinical trials have adopted this approach to incorporate new chemotherapy, radiotherapy and immunotherapy protocols (Figure 1), with the aim of implementing therapeutic schemes that promote effectiveness and safety in each patient.

For this reason, the identification of the molecular group has become a critical component for the clinical approach of MB, emphasizing the importance of diagnostic accuracy, since incorrect assignment of the molecular group would lead to treatment failure; that is, the application of more or less intensive radiotherapy and/or chemotherapy schemes would have a direct impact on the quality and life expectancy of patients.

This paper addresses the different strategies that exist for molecular classification, including their technical and analytical characteristics, as well as the performance of each test, analyzing its classification capacity and accuracy.

## 2. Low-Accuracy Methods for the Molecular Classification of Medulloblastoma

The diagnostic approach for a patient begins with clinical evaluation and imaging studies, such as computed tomography (CT) and magnetic resonance imaging (MRI), followed by safe maximum tumor resection. A pathologist subsequently studies the resected tissue to obtain a definitive diagnosis, performs molecular classification for risk stratification, and finally makes a therapeutic decision.

### 2.1. Molecular Classification of MBs on the Basis of Radiomic Characteristics

In the presurgical stage, different research groups have developed preliminary molecular classification methods, such as location, contrast medium uptake, presence of edema, hemorrhage or metastasis, which are related to the molecular group, on the basis of imaging characteristics [18,19,20]. Although the results are not conclusive, owing to its low sensitivity (60–70%) compared with the final biopsy result, in centers where there is no access to molecular classification methods, the radiomic analysis of preoperative studies can aid in determining patient prognosis.

### 2.2. Molecular Classification of MB by Immunohistochemistry

Once the tumor has been routinely resected, it is placed in formaldehyde for subsequent inclusion in paraffin and histopathological studies, which are essential in classical pathology. One of the most widely used techniques for classifying medulloblastomas is immunohistochemistry (IHC). With this strategy, it is possible to identify the MB SHH and WNT; however, its major limitation is that it cannot distinguish between G3 and G4, encompassing them in a single group called Non-WNT/Non-SHH.

Different panels of between 2 and 8 antibodies have been used with this technique, with the set of 3 targets proposed by Ellison (YAP1, GAB1 and nuclear β-catenin, Table 1) having the best accuracy (93–95%) [7,15,21,22]. However, for 5–7% of cases, the immunoreactivity can be nonspecific; thus, the molecular type cannot be defined [7,15,21]. Notably, when the results obtained by IHC were compared with the methods of classification by massive analysis, agreement between 75% and 95% was detected in the cases analyzed [8,15]. The percentage of concordance with the results of other panels, such as NanoString, ranged from 58% to 92%, where the main inconsistencies were the changes in the SHH and WNT groups (3%), SHH and Non-WNT/Non-SHH groups (3%), and WNT and Non-WNT/Non-SHH groups (4%) [7,8,23].

Despite the fact that the classification by IHC is characterized by its technical and economic accessibility, it also presents methodological limitations such as the variability of antibodies between brands and lots, institutional differences on the basis of tissue fixation and embedding methods, and inter- and intraobserver reproducibility, which are factors that directly influence the accuracy of the test. For example, multiple studies considered different cutoff points for nuclear β-catenin positivity, ranging from 1% to 50% of the tumor area, which could generate disagreement for the identification of MB WNT [7,15,21,23,24,25,26,27]. In addition, inconsistent expression patterns have been observed for the SHH molecular group; for example, SHH tumors are positive for nuclear β-catenin (20% of the tumor area), SHH tumors are negative for GAB1 in up to 6% of cases, and WNT tumors lack nuclear β-catenin expression (associated with necrosis) [22,23]. In addition, indeterminate-group medulloblastomas (YAP1 positive, GAB1 negative and cytoplasmic β-catenin) are associated with the intrinsic expression of YAP1 in melanocytes and muscle cells, which is present in medulloblastomas with myogenic or melanocytic differentiation [28].

Although less frequent, there are also cases where other types of neoplasms that may be erroneously identified as medulloblastomas are immunoreactive to the markers. These include embryonic tumors with multilayer rosettes and atypical rhabdoid teratoid tumors that are positive for YAP1 and cytoplasmic β-catenin [29]. Together, these phenomena can compromise the reliability of the test, so validating the classification result via another methodology would be the most appropriate.

### 2.3. Molecular Classification of MB by Mutation Profiles

Another approach that is particularly useful for the identification of WNT and SHH tumors is the analysis of mutational profiles via genome, exome or targeted sequencing. WNT MBs are identified via these methodologies since more than 90% of these tumors present mutations in CTNNB1, whereas SHH tumors have mutations in genes that encode effector proteins of the pathway, such as PTCH1, SUFU and SMO, which are observed in half of the cases. However, to classify G3 and G4 tumors, this approach is not useful since mutations with sufficient sensitivity (<15%) are not identified to designate either of these two molecular groups [14,30,31,32,33,34]. Therefore, mutational signatures are not the most suitable method for the assignment of the four molecular groups; their utility lies in verifying WNT cases via a CTNNB1-targeted approach. Similarly, identifying the mutational status of p53 in SHH tumors is a factor associated with poor prognosis in patients in this molecular group [8,35].

## 3. Molecular Classification Methods for Medulloblastoma Based on Expression Panels

The methods that can distinguish the four molecular groups with moderate sensitivity are based on tumor expression characteristics. These expression patterns are quantified via two techniques: an amplification-free hybridization system, called NanoString, and polymerase chain reaction (PCR).

### 3.1. NanoString

Derived from omic studies of medulloblastoma, various research groups have sought to reduce the signatures of the four molecular groups. The work of Northcott and colleagues, who defined a panel of 25 genes to carry out molecular classification, stands out (Table 2) [36]. This set of genes is interrogated with the NanoString nCounter platform, which is based on multiplex determination through hybridization between the biological sample and fluorescence-labeled probes contained in a chip, which is free of amplification. The fluorescence signals are associated with the expression of the markers that make up the panel, generating a specific expression profile for each molecular group.

NanoString can identify molecular groups in 90–98% of patients, with accuracies ranging from 67 to 96%, when the results are compared with those obtained with classification methods in massive analyses [7,8,23,36,37]. Among the factors that contribute to the low accuracy, Kavneet revealed that when analyzing RNA obtained from frozen tissue, a correlation of 98% was obtained; in contrast, when RNA isolated from tissue embedded in paraffin was analyzed, the correlation decreased to 67–87%, which was dependent on the age of the paraffin-embedded sample [8]. Importantly, this methodology has limitations, since the presence of false positives and false negatives has been observed for molecular groups, for example, the change in molecular groups between SHH and Group 3/Group 4 (3%), Group 3 and Group 4 (5–8%), and WNT and SHH (3.5%) [8,10,36,38].

Among the technical characteristics of NanoString, the following stands out: (1) it requires highly specialized, high-cost equipment that includes hybridization and counting of the fluorescence signals, which increases the cost of the technique, and (2) the chips that contain the probes are designed to process a minimum of 12 samples simultaneously; if the chip is not used in its entirety, water must be placed; otherwise, the process could fail for the rest of the samples to be analyzed. This last point represents a limitation, since, for most care centers, collecting 12 samples requires more time than the patient can wait between surgery and the start of treatment. For example, for the National Institute of Pediatrics, one of the national reference centers in Mexico, collecting 12 samples would take at least one year.

### 3.2. Quantitative PCR

To mitigate the limitations of NanoString, in 2020, Cruzeiro and colleagues evaluated the same panel of genes via an array of Taqman probes and the relative quantification method via quantitative PCR (RT-PCRq). With this strategy, 97% concordance was obtained with the results of massive analyses, with G3 medulloblastomas showing the lowest precision (88.9%). In an effort to reduce the cost of the panel due to the use of multiple probes, they limited themselves to six genes (Table 2) to classify them into three groups: WNT, SHH and G3/G4 (Non-WNT/Non-SHH; agreement was obtained in all the WNT cases and 86% of the SHH cases) [39].

Similarly, another research group proposed reducing the panel analyzed with NanoString to a set of six genes (Table 2) analyzed by RT-PCR [40]. With this approach, a similar precision was obtained when the entire panel was evaluated. However, the validation cohort was small and lacked WNT cases, so this strategy should be tested in larger cohorts, and it should also include all molecular groups. to define the cutoff points for the expression levels of each marker and to determine the sensitivity and specificity of the test.

Another panel that was proposed for the classification of medulloblastomas is that of Kunder and colleagues, who defined a set of 12 genes and 9 microRNAs analyzed via RT-PCRq [41]. Using this method, up to 90% of the patients can be classified, with 97% accuracy and a 3% exchange of the SHH group with Group 3, taking the results obtained with NanoString as a reference. However, some patients are not classified with this panel, and the majority (~90%) are in Group 3 or Group 4 [21,41].

Methodologically, some of the drawbacks of the use of qPCR panels are that multiple analyses are needed to determine the molecular group of a single sample, which is impractical, in addition to having a high-quality sample (tumor RNA) and quantity that is sufficient for all tests. However, this approach could be one of the simplest to implement in hospitals, as it is an affordable technique in terms of cost, technology, and analysis.

## 4. High-Precision Methods for the Molecular Classification of Medulloblastoma

The methods with the highest sensitivity and specificity are massive analyses at the transcriptional or epigenetic level. Both methods are considered references and are used in clinical practice as well as in research protocols. Among the advantages they share, it is noteworthy that some of these methods have been used to classify larger cohorts, which facilitates the bioinformatic analysis of the results and, consequently, their implementation in new health centers.

### 4.1. Molecular Classification of MB on the Basis of Epigenetic Changes

Two techniques have been used for the epigenetic analysis of MB: microarrays and genome sequencing [6,42]. At a technical level, the main advantage in determining molecular groups with methylation profiles is the nature of the sample, since compared with RNA, DNA is more stable. In addition to the accessibility of nucleic acids, since DNA can be obtained from tissue embedded in paraffin, this approach is favorable since biopsy and its inclusion in paraffin is a routine step in clinical diagnosis. Moreover, to obtain high-quality RNA, it is necessary to extract it from fresh tissue, which is often unavailable because tissues obtained during surgery are immediately stored in formaldehyde, which affects the integrity of the RNA.

#### 4.1.1. Methylation Microarrays

Methylation microarray classification involves detecting methylated DNA regions by hybridizing bisulfite-treated samples with probes designed to target specific genomic sites prone to methylation, including CpG islands, gene bodies, intergenic regions, and promoters.

Three Illumina platforms were used for medulloblastoma studies: GoldenGate Cancer Panel I, 450K/EPIC Human Infinium and the 850K/EPIC Human Infinium chip, which interrogate more than 1505, 450 thousand and 850 thousand methylation sites, respectively. Among these platforms, the most widely used is the 450K chip, with which molecular assignment results have been obtained for 95–100% of the patients questioned [10,15,38,39,43,44]. For 2–4% of patients, the analysis could not be performed because the sample size was insufficient [15,45].

When the percentage of correspondence between the results of classification by microarrays of expression and methylation was compared, it was observed that all the WNT and SHH cases were correlated by both methodologies. However, for Group 3 and Group 4, false positives were observed in 5% of the cases (Table 3) [46]. Among the factors that could explain the inconsistency of the results are their molecular similarity or that it is an intermediate molecular lineage between both groups, as described by Williamson in 2022 [44]. To resolve this discrepancy, complementary analysis of other clinical (age and sex) and biological characteristics (histological type and genetic alterations) may help to discern the molecular type. For example, large cell histology and MYC amplification are predominant in MB-Group 3 but rare in MB-Group 4, and vice versa, MYCN amplification is predominant in MB-Group 4 in comparison to Group 3 [44,47,48,49].

The most relevant advantage of methylation microarrays over other classification methods is their ability to distinguish other types of tumors, such as ependymomas, glioblastomas or astrocytomas neoplasms, that have been erroneously diagnosed as medulloblastomas by histopathology, representing up to 8% of patients [38,45]. Therefore, some authors refer to this methodology as the gold standard for the assignment of the histological lineage [8,38]. The practical limitations of classification using this method include the fact that the most widely used platform (Infinium, Illumina), requires the processing of at least eight samples simultaneously, which increases the cost of analyzing individual samples or causes delays the collection of enough samples to fill the cartridge, which in turn hinders timely molecular diagnosis and personalized treatment of patients.

#### 4.1.2. DNA Sequencing

An alternative strategy for obtaining methylation profiles is genome sequencing. Two platforms have been used in the study of medulloblastoma: Illumina and, recently, Oxford Nanopore Technologies (ONT) [37,50]. As with methylation microarrays, with Illumina sequencing, the tumor DNA sample must be pretreated with sodium bisulfite to distinguish methylated bases. With ONT technology, no prior treatment is required since the platform allows direct DNA sequencing, eliminating the risk of degradation due to bisulfite conversion and bias due to the amplification used in the construction of the Illumina libraries [37].

In addition to the methylome, ONT platforms allow other genomic data, such as small insertions and deletions (indels), structural variants, fusion genes, single nucleotide variations (SNVs) and copy number varintions (CNVs), to be studied. These last two alterations are relevant for the detection of MYC amplification and the identification of TP53 mutations, which characterize patients with high-risk MB-Group 3 and MB-SHH, respectively [51,52,53]. Another advantage of this technology is its capability to perform real-time sequencing of specific genomic regions through adaptive sampling, thereby eliminating the need for additional enrichment steps during library preparation. This approach enables the rapid generation of molecular classification results, often within only a few hours of sequencing with >90% concordance compared with results from methylation arrays [54,55,56].

The incorporation of this recent sequencing technology into the classification of central nervous system (CNS) tumors has facilitated the expansion and validation of reference datasets used by various classifiers, such as rapid-CNS [54,55,57]. This advancement enhances the robustness of existing models and promotes the adoption of these diagnostic technologies in new clinical centers. Furthermore, standardized reporting formats have been proposed that integrate information from sample preprocessing, such as amount and purity (percentage of tumor cells), with details of the classification models and confidence scores [55,58,59].

Methylome sequencing classification can identify molecular groups in up to 98% of patients, with a mismatch between Group 3 and Group 4 (5% of patients) [37,50]. As it is a recent approach and has been demonstrated in small cohorts of patients with medulloblastomas, the results could be influenced by the size of the sample, since the analysis methods are based on learning models [50,56,60]. The accuracy of the classification model is dependent on the reference used to train it, so it is important to consider the number of samples in the sets as well as the number and names of classes (molecular types) considered for the classification. These limitations can be offset by collaborative processes for data sharing and harmonization of the classes [58,61]. Furthermore, owing to the inherent heterogeneity of medulloblastoma tumors, it is very likely that in populations around the world, cases with intermediate phenotypes will continue to be identified, leading to discrepancies in molecular assignment.

#### 4.1.3. Minimal Methylation Classifier (MIMIC)

Another resource used in classification by methylation patterns is the identification of a methylome signature that includes 17 methylation regions (CpG loci) called MIMIC. To design this method, the most variable regions of methylation among the four molecular groups were extracted from 450K/EPIC Human Infinium microarray data and analyzed with Agena iPLEX technology, which is based on PCR and mass spectrometry (MS).

The DNA sample is previously subjected to bisulfite to distinguish the methylated cytosines, and the regions of interest are amplified via PCR to later extend a single nucleotide such that a nucleotide is incorporated into a methylated region (retaining the cytosine) of guanine; otherwise, in an unmethylated region (converted to uracil), a thymine nucleotide is added. Finally, the amplification products are analyzed by MS to determine the methylation status on the basis of molecular weight. With this approach, the accuracy is between 92% and 98% [62]. However, its application has not been adopted in healthcare centers since it is not widely accessible because of the cost of the MS platform.

### 4.2. Molecular Classification of MB on the Basis of the Transcriptome

Transcriptomic analyses of medulloblastoma were the first to reveal the presence of these four molecular groups. Therefore, in 2011, Northcott published a work in which the molecular consensus of MB was established; each group was named on the basis of its expression characteristics, except for G3 and G4, which lack specific pathways [63]. Molecular classification via expression microarrays is one of the preferred strategies because of its high sensitivity and specificity.

#### Microarrays of Expression Data

The first evidence of the existence of the four molecular groups of MB was through the analysis of expression profiles via the HumanGene U133 microarray platform (versions A and U133 Plus 2.0) [64,65,66]. Other arrays, such as Human Exon 1.0, Human Gene 1.1 ST, Human Gene 2.0 ST and Whole Human Genome 4 × 44 K, were subsequently used [6,42,67,68]. These platforms differ in the array density, number and regions of the transcripts that they identify; for example, the Human Gene 2.0 ST chip contains 1.35 million probes that identify the expression of 418 thousand exons and 48 thousand markers for the 3′ regions of the messenger RNAs (mRNAs), whereas the Human Exon 1.0 array has approximately 5.5 million probes distributed in more than one million exons, which represent all the transcripts recorded in 2009 (Hg19).

However, regardless of the type of arrangement and bioinformatic analysis used to interpret the results (principal component analysis, nonnegative matrix factorization or hierarchical clustering), all patients are classified; that is, there are no ambiguous cases, as this methodology has been considered the gold standard for the molecular classification of the MB, that is, with a precision of 100% [30,34,46,67]. The largest cohorts worldwide have been classified with this methodology, representing an advantage for its implementation in new centers since it facilitates the training and validation of the analysis models.

Likewise, among the aspects to consider in massive analyses are the technical requirements and infrastructure, which are key factors, since at least three highly specialized pieces of equipment need to be classified by microarrays: the hybridization chamber, the fluid station and the scanner. Moreover, the technical complexity of the processing requires trained personnel to maintain the integrity of the sample throughout the process, as well as personnel with specialized training for the bioinformatic analysis of the results. This represents a challenge for its implementation in clinical practice, especially for healthcare centers in low- and middle-income countries.

## 5. Conclusions and Perspectives for the Molecular Classification of Medulloblastoma

Medulloblastoma is the embryonic tumor with the highest morbidity and mortality in children. It is characterized by high intra- and intertumor heterogeneity, which is why its omic study revealed the existence of four molecular groups with biological, clinical and prognostic differences. Currently, comprehensive classification of medulloblastoma, supported by the molecular group, is the best way to stratify and treat patients. Therefore, accurate classification is essential in current clinical practice.

There are several classification methods that differ in diagnostic capacity, sensitivity and specificity, with those based on radiomic characteristics, mutation profiles and IHC being the least accurate. These are followed by expression panel-based methods such as NanoString. The most accurate methods are massive gene expression analyses and DNA methylation profiling.

Currently, the choice of a molecular classification method mainly depends on the availability of resources; however, it is necessary to develop a standard guide based on multicenter studies that allows evaluation of the influence of other factors associated with the processes (batches of antibodies, nucleic acid extraction methods, bioinformatics methods, among others) on the reproducibility, sensitivity, and specificity of each test.

This review shows the capacity of the various methods to identify WNT and SHH medulloblastomas, which, even with basic techniques such as IHC, are highly sensitive, in contrast to Group 3 and Group 4 tumors, for which even with the most robust techniques, there are unclassified cases; thus, the results are inconsistent compared with those of other methodologies. It is very likely that the high heterogeneity of the neoplasm leads to ambiguous cases (not classifiable), so combining classification strategies and verifying the results via a second method, or even a third method, would be the most reliable approach.

Some authors have proposed workflows that include more than one molecular classification strategy [8], which are useful for centers that have the necessary human and technological resources. However, for developing countries, most methods are inaccessible, with IHC being the most widely adopted technique [25,26,27]. G3 and G4 tumors, which differ in risk and prognosis, are uncertain.

The implementation of molecular tests that identify the four molecular groups with high precision clearly requires a high infrastructure capacity; trained technical personnel; and a multidisciplinary team (molecular biology, histopathology, molecular pathology, pediatric neurosurgery, radiology and oncology) trained to generate, interpret and transfer the results to therapy. The addition of other factors, such as sample collection and preservation methods, represents a challenge in clinical practice, since all this must occur in a period of four weeks, the recommended time between postsurgical recovery and the start of treatment.

These factors present a challenge for their implementation, especially for healthcare centers in low- and middle-income countries, such as ours. This gap could be overcome with the incorporation of new technologies, such as ONT platforms, since they are robust methodologies that are more economically and operationally accessible, which would have a positive impact on molecular pathology. In addition, the study of other molecular characteristics, such as alternative transcripts or epitranscriptomic profiles, could lead to new classification methods.

## Figures and Tables

**Table 1:** Immunoreactivity of the Ellison panel for the molecular classification of MB by IHC.

Antibody	WNT	SHH	Non-WNT/Non-SHH
Β-Catenin	Positive N	Negative N Positive C and/or M	Negative
YAP1	Negative	Positive	Negative
GAB1	Negative/positive N and M	Positive	Negative

N: nuclear; C: cytoplasmic; M: membrane.

**Table 2:** Genes analyzed with NanoString for the molecular classification of medulloblastoma.

Molecular Group	Expression Signature
WNT	**WIFI1** *, TNC, GAD1, **DKK2**, **EMX2** *
SHH	PDLIM3, **EYA1**, **HHIP**, ATOH1, **SFRP1**
Group 3	IMPG2 *, GABRA5, EGFL11, NRL, MAB21L2, NPR3 *
Group 4	KCNA1, EOMES, KHGRBS2 *, RBM24 *, UNC5D, OAS1
Constitutive expression genes	**ACTB, GAPDH, LDHA**

The reduced panel proposed by Cruzeiro is indicated in bold. The symbol * represents the genes that make up the panel proposed by Gershanov and colleagues.

**Table 3:** Characteristics of the methods for molecular classification of MB. A comparative table of the main molecular classification methods is shown. Accuracy data were obtained by comparing the results against the reference method (expression microarrays). ONT: Oxford Nanopore sequencing. * Except for ONT platforms.

Method	IHC	Panel RT-PCRQ	Nanostring	DNA Sequencing	Methylation Array	Expression Array
**Accuracy (%)**	75–95	86–95	80–90	>95	95–100	100
**Unclassified (%)**	7	No reported	10	2	5	0
**Group exchange (%)**	6% SHH- Non-WNT/Non-SHH	3% G3-SHH	8–12% G3–G4 5% G3-SHH 6% G4-SHH	5% G3–G4	5% G3–G4	0
**Pros**		Classification into four groups				
	Economic and technological accessibility			Discriminates other types of tumors		
				ONT: native DNA reading, processing from one to 48 samples.		
**Cons**	Cannot distinguish between G3 and G4	Multiple experiments	High infrastructure requirements *			
	Intra and inter-observer variations	High-quality and quantity of sample	Minimum 12 samples	Illumina platforms require a minimum of samples (8 for microarrays).		High-quality sample
				Illumina platforms require sample pretreatment that could lead to bias or degradation.		
**Cost per sample (US$)**	40–70	70	110 Equipment: 300,000	Illumina platforms: 700 ONT: 876 (up to 48 samples)	1600	1600
**Turnaround time (days)**	3–2	5–4	7	Illumina platforms: >14 ONT: 1–3	11–7	7

## Data Availability

No new data were created or analyzed in this study. Data sharing is not applicable to this article.
